# Inbreeding depression for kit survival at birth in a rabbit population under long-term selection

**DOI:** 10.1186/s12711-020-00557-3

**Published:** 2020-07-08

**Authors:** Ino Curik, György Kövér, János Farkas, Zsolt Szendrő, Róbert Romvári, Johann Sölkner, Istvan Nagy

**Affiliations:** 1 Department of Animal Science, Faculty of Agriculture, University of Zagreb, Zagreb, Croatia; 2 Institute of Methodology, Faculty of Economic Science, Kaposvár University, Kaposvár, Hungary; 3 Institute of Animal Science, Faculty of Agricultural and Environmental Sciences, Kaposvár University, Kaposvár, Hungary; 4 Division of Livestock Sciences, University of Natural Resources and Applied Life Sciences, Vienna, Austria

## Abstract

**Background:**

Accumulation of detrimental mutations in small populations leads to inbreeding depression of fitness traits and a higher frequency of genetic defects, thus increasing risk of extinction. Our objective was to quantify the magnitude of inbreeding depression for survival at birth, in a closed rabbit population under long-term selection.

**Methods:**

We used an information theory-based approach and multi-model inference to estimate inbreeding depression and its purging with respect to the trait ‘kit survival at birth’ over a 25-year period in a closed population of Pannon White rabbits, by analysing 22,718 kindling records. Generalised linear mixed models based on the logit link function were applied, which take polygenic random effects into account.

**Results:**

Our results indicated that inbreeding depression occurred during the period 1992–1997, based on significant estimates of the z-standardised classical inbreeding coefficient z.F_L_ (CI_95%_ − 0.12 to − 0.03) and of the new inbreeding coefficient of the litter z.F_NEWL_ (CI_95%_ − 0.13 to − 0.04) as well as a 59.2% reduction in contributing founders. Inbreeding depression disappeared during the periods 1997–2007 and 2007–2017. For the period 1992–1997, the best model resulted in a significantly negative standardised estimate of the new inbreeding coefficient of the litter and a significantly positive standardised estimate of Kalinowski’s ancestral inbreeding coefficient of the litter (CI_95%_ 0.01 to 0.17), which indicated purging of detrimental load. Kindling season and parity had effects on survival at birth that differed across the three periods of 1992–1997, 1997–2007 and 2007–2017.

**Conclusions:**

Our results support the existence of inbreeding depression and its purging with respect to kit survival at birth in this Pannon White rabbit population. However, we were unable to exclude possible confounding from the effects of parity and potentially other environmental factors during the study period, thus our results need to be extended and confirmed in other populations.

## Background

Inbreeding cannot be avoided in small closed populations and can lead to inbreeding depression (ID), which is defined as a reduction of the population mean for quantitative traits such as reproduction, fecundity or survival traits, as well as other traits. Inbreeding depression has been documented in wild animals [1, 2], animals in captivity [3], laboratory animals [4], domesticated animals [5], and humans [6]. This important evolutionary force threatens the survival of genetically small populations. Although several theories have been developed to explain various aspects of ID [7–9], several questions remain open.

One important question is whether ID can be “purged” or reversed through the interaction of inbreeding and selection [7, 10]. Purging of detrimental load is based on the idea that inbreeding increases the frequency of rare deleterious alleles such that they appear more often in homozygous states, on which natural and/or artificial selection can act to remove them more efficiently. To what extent purging occurs among mammals in nature is unclear. Templeton and Read [10, 11] reported purging of ID in a small captive population of Speke’s gazelles within a few generations by mating related individuals. However, reassessment of the statistical methods used in that study suggested that the observed ID purging did not result from the planned breeding strategy [12–14]. In a study of 25 captive mammalian populations, Ballou [3] reported ID purging but concluded that the effects were too weak to be practical as a general strategy for eliminating ID. Similarly, a meta-analysis of 119 zoo animal populations concluded that ID purging exerts negligible effects on the frequency of deleterious alleles [15]. In some species (e.g. cattle), the recent introduction of genomics has intensified artificial selection and consequently accelerated an increase in homozygosity and accumulation of detrimental load [16, 17]. This has increased interest in the question of whether purging can be achieved practically, since breeders seek to reduce detrimental load in animal populations.

To evaluate ID purging, Ballou [3] proposed the ancestral inbreeding coefficient (F_A-B_), which is defined as the probability that any allele in an individual has been autozygous at least once in previous generations [18]. In the applied logistic regression model, negative impacts of inbreeding coefficients (β_F_) and positive interaction effects between inbreeding coefficients and ancestral inbreeding coefficients (ß_FFA-B_) on neonatal survival and litter size indicated ID purging [3]. When purging involves only mildly deleterious alleles, a slightly different model (ancestral inbreeding coefficients are used instead of the interaction term) can detect it with more sensitivity [19]. In a stochastic approach that quantitates the effects of ID purging, Kalinowski et al. [14] decomposed the calculation of an individual’s inbreeding coefficient into a ‘new’ or ‘recent’ inbreeding coefficient (F_NEW_) and an ‘old’ or ‘remote’ inbreeding coefficient (F_A-K_). Thus, F_NEW_ is defined as the probability of autozygosity for an allele, which was not present in autozygous state in previous generations, whereas F_A-K_ is defined as the probability that any allele in an individual is currently autozygous and has been autozygous at least once in previous generations [18]. A third approach is based on the reasoning that the amount of purging depends on the cumulative autozygosity of all ancestors, since in the same pedigree path two ancestors should not be homozygous for the same deleterious allele. This method is called “expressed opportunity of purging” and can be applied only to purging that occurs within a few generations, more details are in Gulisija and Crow [20]. Conversely, a fourth approach, called the “inbreeding-purging method”, can be applied to purging that occurs over a long time [21]. According to López-Cortegano et al. [22], the “inbreeding-purging method is based on a purged inbreeding coefficient that predicts how mean fitness and inbreeding load are expected to evolve in a population undergoing inbreeding”. It is still not clear which of these approaches is more effective in detecting purging. Simulations suggest that both the approach based on the ancestral inbreeding coefficient [3, 19] and the inbreeding-purging method [21] give biased estimates of ID but that the latter can detect the presence of purging with more sensitivity [22]. However, the inbreeding-purging method does not consider random polygenic additive effects, which can strongly bias ID estimates [23, 24]. Random polygenic effects have long been used in animal breeding through the ‘individual animal model’ [25].

Regardless of the approach used, studies to analyse ID purging have generally neglected potential confounding due to environmental variation, such as changes in food resources, climate, husbandry, and exposure to parasites or pathogens. These factors can exert a form of natural and/or artificial selection that alters the magnitude of ID and may therefore confound analysis of purging [14]. Thus, in long-term experiments, improvement of the environment can decrease ID and consequently could be misinterpreted as evidence of purging [26–28]. How to reduce the impact of such confounding on the identification of purging is not straightforward, since alleles that interact with these environmental factors may themselves be purged if many generations are under analysis [29].

**Fig. 1:**
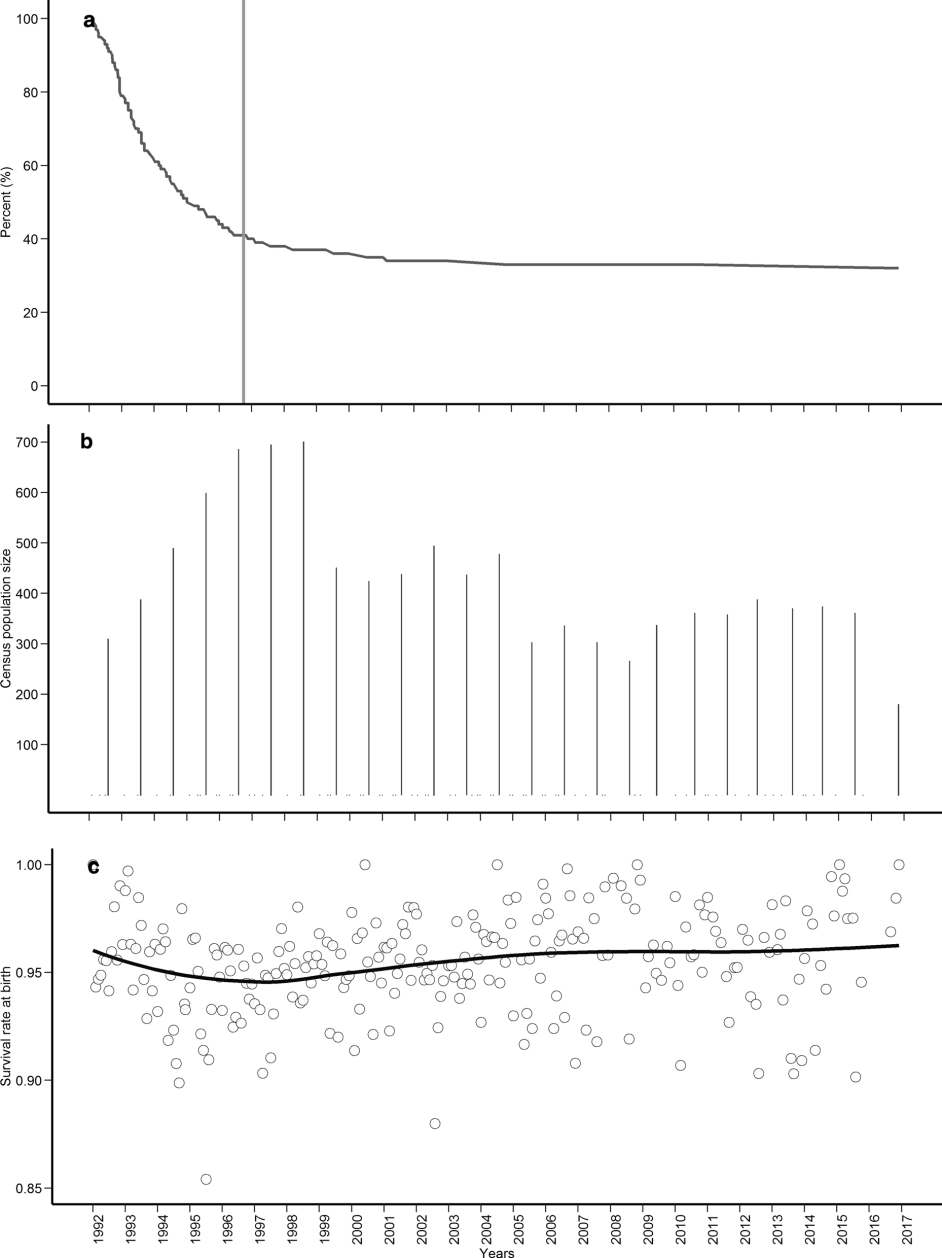
Trends in the contribution of founders to the population (**a**), in census size (**b**), and in survival rate of kits at birth (**c**) in the breeding program of Pannon White rabbits from December 1992 to November 2017

In this study, our aim was to quantify the magnitude of ID for birth survival rate in a population of Pannon White rabbits (Fig. 1) and identify signs of its purging. This population is well suited to inbreeding studies because it forms a closed population that has been under selection in a relatively controlled environment, without immigration, and accurate pedigrees are available for many generations. In a previous study, the effect of inbreeding was estimated for traits related to litter size [30] using dominance animal models. In the current study, we applied an approach based on information theory and multi-model inference, and accounted for a polygenic component and the binary nature of the birth survival trait.

## Methods

### Population foundation and management

The Pannon White rabbit breed was developed at Kaposvár University (Kaposvár, Hungary) in the late 1980s and was recognised as a rabbit breed in 1992. It has been selected as a closed population ever since. Since 2001, selection of this rabbit breed is based on in vivo computer tomography measurements, and it has been exploited extensively for meat production. Over time, the traits under selection have changed several times, i.e.: average daily gain from 1992 to 2000; average daily gain, L-value from 2000 to 2003, average daily gain, thigh muscle from 2004 to 2010, and litter weight at day 21, thigh muscle volume since 2010.

### Data and pedigree information

Analysis was based on the pedigree data and kindling records (n = 22,718) that were collected continuously from 1992 to 2017. To calculate the inbreeding coefficients of the litters, dummy progenies were created according to the unique combinations of does and related mating bucks. The extended pedigree with dummy progenies included 29,802 individuals that were bred from 1421 bucks and 5339 does.

### Inbreeding coefficients and effective population size

To test our hypotheses, we used several types of inbreeding coefficients. The inbreeding coefficients of the dam (F_D_) and of the litter (F_L_) [31] are defined as the probability that the two alleles at any locus in an individual are identical-by-descent. The Kalinowski “new” inbreeding coefficients of the dam (F_NEWD_) and of the litter (F_NEWL_) [14] are defined as the probability that any allele in an individual is autozygous for the first time. F_L_ (F_D_) can be decomposed into F_NEWL_ (F_NEWD_) and F_A-KL_ (F_A-KD_), where the Kalinowski ancestral inbreeding coefficient is the probability that a currently autozygous allele has been autozygous at least once in previous generations [18]. The ancestral inbreeding coefficients of the dam (F_A-BD_) and litter (F_A-BL_), which were introduced by Ballou [3], were defined by Baumung et al. [18] as the probability that any allele in an individual has been autozygous at least once in previous generations.

When modelling ID in polytocous mammalian species, such as mice [32], pigs [33], rabbits [30] and dogs [34], the negative effects of the mother’s (dam’s) inbreeding on litter-associated traits, probably by influencing uterine capacity, must also be considered. To obtain unbiased inbreeding coefficients, they were calculated by the stochastic method known as “gene dropping”, as implemented in the GRain v2.2 software, available at https://boku.ac.at/nas/nuwi/software, with 10^6^ iterations [18, 35]. A detailed description of the calculation of F_NEW_ and F_A-K_ is provided in Doekes et al. [36]. Complete generation equivalent (CGE_D_ or CGE_L_) was computed as the sum of (1/2)^n^ over all known ancestors, where n is the number of generations separating the individual from the known ancestors, and effective population sizes (Ne) were derived by the approach described in Perez-Enciso [37]. CGE_D_, CGE_L_ and Ne were calculated using the ENDOG v4.8 software [38].

### Statistical analyses and modelling

ID was modelled using an approach that is based on information theory [39–41], which has gained popularity in evolutionary modelling [42, 43], while the effects of inbreeding on kit survival at birth, treated as a binomial response trait, were analysed using generalised linear mixed models (GLMM), which were fitted using the *lme4* [44] and *pedigreemm* [45] packages in R.

Given the exponential increase in computation time as the size of the dataset increased, we did not model the entire breeding period. Instead, we split the data into three consecutive time-periods: December 1992 to August 1997, September 1997 to October 2007, and November 2007 to November 2017. The timing of the first period was set based on the changing trend observed for (a) number of contributing founders, (b) population size, and (c) kit survival at birth (Fig. 1). The lowest value for survival at birth was observed in August 1997 (Fig. 1), and it recovered thereafter. The recovery period was divided into two periods to allow us to control for any confounding due to environmental variations.

#### Defining hypotheses and modelling parameters

We modelled ID using two approaches: (1) Ballou’s approach based on ancestral inbreeding coefficients [3], with Boakes’ adjustment for mildly deleterious alleles [19]; and (2) Kalinowski’s approach based on F decomposition [14]. All statistical models used additive genetic effects, the fixed effects of parity number (categorised as 1, 2, 3–10, or 11+) and kindling season (non-summer vs. summer, which was defined as 15 June to 15 September). Year of kindling was omitted because of strong collinearity with inbreeding coefficients. Results and conclusions derived from the computer simulations [23] and empirical evidence [24] showed that inclusion of the additive relationship matrix is important in the estimation of inbreeding depression when the population is under selection and inbreeding increases. Model input variables were z standardised (denoted “z.*variable*”), by using the *arm* package [46], which facilitates comparison of models.

Both modelling approaches began with the inclusion of the same fixed effects and polygenic additive effects (animal). In the Ballou–Boakes approach, we considered all possible combinations of factors that can be included in the model with respect to the inclusion of the covariates of F_D_, F_L_, and F_A-BL_, along with the fixed effects of season and parity, and the random effect of animal in the model. The most complicated model had the following structure: logit[probability(survival)] = parity + season + z.F_D_ + z.F_L_ + z.F_A-BL_ + animal + residual. This model allowed us to detect ID caused by inbreeding in dams and litters, and to detect ID purging caused by litter inbreeding, as described by Boakes and Wang [19]. To reduce model complexity, we did not include the interaction between inbreeding and ancestral inbreeding coefficients [3].

In the Kalinowski approach, we considered all possible combinations of model elements with respect to the inclusion of z.F_NEWD_, z.F_NEWL_, z.F_A-KL_, season, parity, and animal. The most complicated model had the following structure: logit[probability(survival)] = parity + season + z.F_NEWD_ + z.F_NEWL_ + z.F_A-KL_ + animal + residual. This model allowed us to detect ID caused by new inbreeding of dams and litters, and to detect ID purging [14].

#### Model selection and averaging

We chose the best combination of models to test each of our hypotheses [39, 40]. The best models with the lowest Akaike’s information criteria (ΔAICc = 2) were selected, as implemented in the *MuMIn* package in R [47]. More details about the modelling strategy and criteria for model selection are in [40].

Estimates for the fixed effects from the selected models were averaged using the natural average method [42]. When a given inbreeding coefficient was included in selected models, the regression coefficients were averaged. When selecting the best models, to take uncertainty into account, the estimates from a given model were weighted during averaging by accounting for Akaike weights [39, 40].

## Results

### Inbreeding in the population: description, trends and relationships

**Fig. 2:**
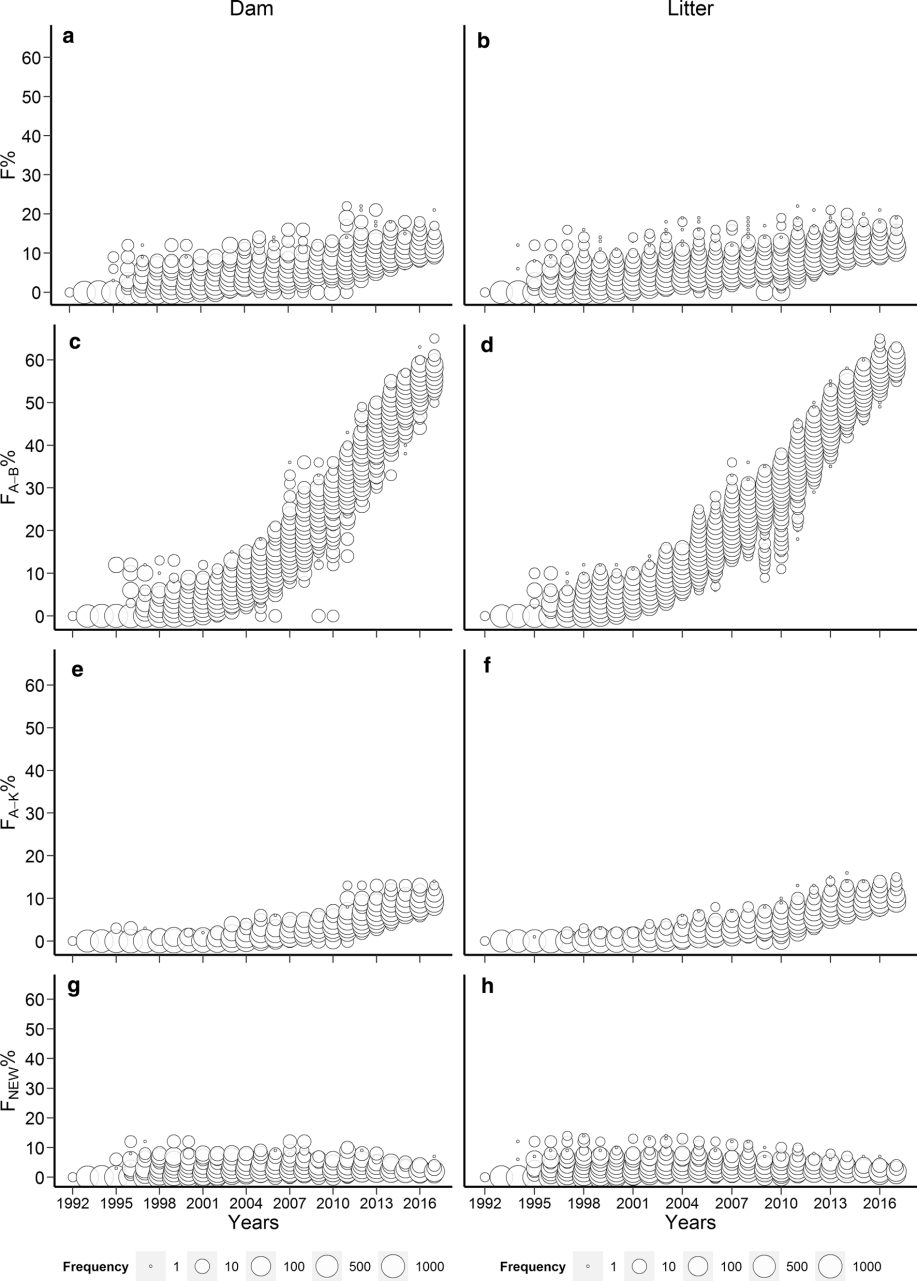
Trends in inbreeding coefficients for dams and litters in the Pannon White rabbit breeding program from December 1992 to November 2017. Inbreeding coefficients for dams (**a**) and litters (**b**), Ballou’s ancestral inbreeding coefficient [3] for dams (**c**) and litters (**d**), Kalinowski’s ancestral inbreeding coefficient [14] for dams (**e**) and litters (**f**), and Kalinowski’s new inbreeding coefficient [14] for dams (**g**) and litters (**h**)

Descriptive statistics of all inbreeding coefficients are in Table 1. Trends and variation in inbreeding coefficients (F, F_NEW_, F_A-B_ and F_A-K_) over 300 months are shown in Fig. 2. The effective population size across all periods was Ne = 67 (ΔF = 0.00746), but it declined over time, from Ne = 115 (ΔF = 0.00436) in the first period to Ne = 78 (ΔF = 0.00642) in the second, and to Ne = 67 (ΔF = 0.00744) in the third period.

**Table 1:** Descriptive statistics of various inbreeding coefficients and complete generation equivalents for the entire study period

Parameter	Mean	Median	Standard deviation	Maximum
F_L_	0.040	0.031	0.039	0.219
F_D_	0.036	0.023	0.034	0.219
F_NEWL_	0.019	0.019	0.016	0.143
F_NEWD_	0.016	0.016	0.016	0.125
F_A_BL_	0.160	0.074	0.185	0.652
F_A_BD_	0.137	0.041	0.182	0.652
F_A_KL_	0.023	0.006	0.031	0.161
F_A_KD_	0.020	0.003	0.030	0.141
CGE_L_	8.976	7.662	5.703	22.07
CGE_D_	7.840	6.032	5.968	21.40

*F*_*L*_ inbreeding coefficient of litters, *F*_*D*_ inbreeding coefficient of dams, *F*_*A-BL*_ ancestral inbreeding coefficient of litters, *F*_*A_BD*_ ancestral inbreeding coefficient of dams, *F*_*NEWL*_ new inbreeding coefficient of litters, *F*_*NEWD*_ new inbreeding coefficient of dams, *F*_*A-KL*_ Kalinowski (ancestral) inbreeding coefficient of litters, *F*_*A_KD*_ Kalinowski (ancestral) inbreeding coefficient of dams, *CGE*_*L*_ complete generation equivalent of litters, *CGE*_*D*_ complete generation equivalent of dams

The ancestral inbreeding coefficients of Ballou and Kalinowski continuously increased over time, especially after 2004. In 2016 and 2017, we observed a high level of ancestral inbreeding, with averages of 0.653 for F_A-BL_ and F_A-BD_ (Fig. 2). This is among the highest reported levels of ancestral inbreeding in mammals, even higher than the level observed for Przewalski’s horses (F_A-BL_ = 0.555) [3], although it is slightly lower than the level observed in inbred laboratory mice [32]. The Kalinowski ancestral inbreeding coefficients reached the maximum values of 0.140 for F_A-KL_ in 2014 and 0.126 for F_A-KD_ in 2017. This implies that the genome of most rabbits had already occurred in an IBD state at least once by the last generation in the study period. The average F_NEWL_ reached a maximum of 0.129 in 2003, whereas the average F_NEWD_ reached a maximum of 0.127 in 2008.

**Fig. 3:**
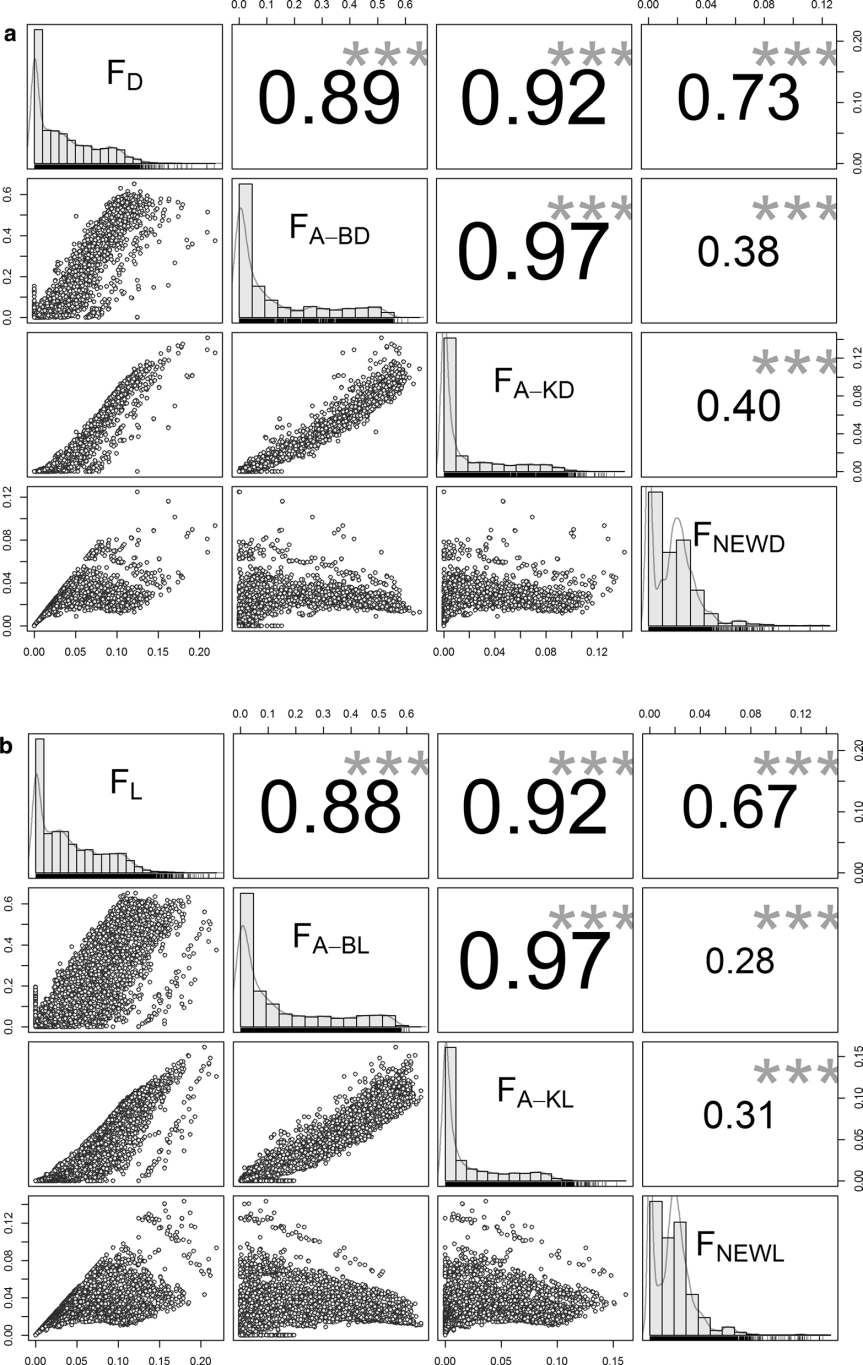
Linear correlations among inbreeding coefficients for dams (**a**) and litters (**b**). F_D_: inbreeding coefficient of dams; F_L_: inbreeding coefficient of litters; F_A-BD_: ancestral inbreeding coefficient of dams; (F_B-AL_): ancestral inbreeding coefficient of litters; F_A-KD_: Kalinowski’s inbreeding coefficient of dams; F_A-KL_: Kalinowski’s inbreeding coefficient of litters; (F_NEWD_): Kalinowski’s „new” inbreeding coefficient of dams; F_NEWL_: Kalinowski’s „new” inbreeding coefficient of litters

The Pearson correlation coefficients between the various dam and litter inbreeding coefficients are provided in Fig. 3. Stronger correlations were obtained between classical and ancestral inbreeding coefficients than between ancestral and new inbreeding coefficients. Extremely strong correlations were obtained between the Ballou (F_A-B_) and the Kalinowski (F_A-K_) ancestral inbreeding coefficients of the dams and of the litters.

### Environmental and additive polygenic effects

The results of the model selection performed over the three breeding periods are in Table 2, which shows only the top models that satisfied the 2AIC_C_ criterion. All selected models included the effects of season, parity, and animal, which confirmed the need to model these effects for kit survival at birth (Table 2). The relative importance of the season was equal to 1 for all models, whereas the estimated effects of season were significant and negative, indicating that summer was less favourable than the other seasons (Tables 3 and 4). Parity number influenced the survival rate of kits at birth significantly in all periods and models. Thus, with few exceptions, the survival of kits at birth was significantly higher in the second and later parities than in the first parity (Tables 3 and 4), and the relative importance was equal to 1 for all analyses (Tables 3 and 4). All selected models also included animal genetic effects, suggesting that their inclusion is essential for an accurate estimation of ID over a long period.

**Table 2:** Properties of models selected according to the ΔAICC ≤ 2 criterion in two modelling approaches to estimate inbreeding depression for survival rate of Pannon White kits at birth in three time periods

Model components	Period								
	Dec 1992 to Aug 1997			Sept 1997 to Oct 2007			Nov 2007 to Nov 2017		
	AIC_C_	Weight	ΔAICc	AIC_C_	Weight	ΔAICc	AIC_C_	Weight	ΔAICc
Ballou-Boakes modelling approach									
Parity + season + animal	x	x	x	*22,428.8*	*0.29*	*0.0*	12,098.1	0.33	*0.0*
Parity + season + z.F_L_ + animal	*9684.8*	*0.49*	*0.0*	22,430.0	0.16	1.2	*12,098.1*	*0.33*	*0.0*
Parity + season + z.F_D_ + animal	x	x	x	22,428.9	0.28	0.1	X	x	x
Parity + season + z.F_A-BL_ + animal	x	x	x	22,430.6	0.1	1.7	X	x	x
Parity + season + z.F_L_ + z.F_D_ + animal	9686.7	0.19	1.9	22,430.0	0.16	1.2	12,100.1	0.1	2.0
Parity + season + z.F_L_ + z.F_A-BL_ + animal	9685.7	0.31	0.9	x	x	x	12,098.9	0.22	0.8
Kalinowski modelling approach									
Parity + season + animal	x	x	x	*22,428.8*	*0.23*	*0.0*	*12,098.1*	*0.32*	*0.0*
Parity + season + z.F_NEWL_ + animal	x	x	x	22,430.3	0.11	1.5	12,098.6	0.26	0.5
Parity + season + z.F_NEWD_ + animal	x	x	x	22,429.2	0.19	0.4	12,100.1	0.12	1.9
Parity + season + z.F_A-KL_ + animal	x	x	x	22,429.1	0.20	0.3	12,098.2	0.30	0.1
Parity + season + z.F_NEWL_ + z.F_NEWD_ + animal	x	x	x	22,430.8	0.09	2.0	x	x	x
Parity + season + z.F_NEWL_ + z.F_A-KL_ + animal	*9680.0*	*0.73*	*0.0*	x	x	x	x	x	x
Parity + season + z.F_NEWD_ + z.F_A-KL_ + animal	x	x	x	22,429.2	0.19	0.4	x	x	x
Parity + season + z.F_NEWL_ + z.F_NEWD_ + z.F_A-KL_ + animal	9682.0	0.27	2.0	x	x	x	x	x	x

ΔAICc reflects the difference among selected models, values for the best model are indicated in italics

*Parity* parity order of kindling, *season*: season of kindling, *z.F*_*L*_: effect of the standardised inbreeding coefficient of the litter, *z.F*_*A-BL*_: standardised ancestral inbreeding coefficient of the litter, *z.F*_*NEWL*_: standardised new inbreeding coefficient of the litter, *z.F*_*NEWD*_: standardised new inbreeding coefficient of dams, *z.F*_*A-KL*_: standardised Kalinowski (ancestral) inbreeding coefficient of the litter

**Table 3:** Standardised estimates of effects on survival rate of kits at birth after averaging of models that satisfy the ΔAICc ≤ 2.0 criterion in the Ballou-Boakes modelling approach

Variable	Dec 1992 to Aug 1997		Sept 1997 to Oct 2007		Nov 2007 to Nov 2017	
	Estimate (95% CI)	RI	Estimate (95% CI)	RI	Estimate (95% CI)	RI
Intercept	3.20 (2.99; 3.40)		2.80 (2.41; 3.18)		3.79 (2.71; 4.86)	
z.F_L_	− *0.08* (− *0.12;* − *0.03*)	1.00	0.02 (− 0.02; 0.06)	0.31	0.05 (− 0.02; 0.12)	0.67
z.F_D_	0.01 (− 0.07; 0.08)	0.19	− 0.05 (− 0.12; 0.02)	0.43	− 0.03 (− 0.05; 0.04)	0.12
z.F_A-BL_	0.04 (− 0.04; 0.13)	0.31	− 0.04 (− 0.14; 0.04)	0.12	− 0.14 (− 0.17; 0.11)	0.22
Season	− *0.25* (− *0.35;* − *0.15*)	1.00	− *0.12* (− *0.19;* − *0.05***)**	1.00	− *0.61* (− *0.71;* − *0.53*)	1.00
Parity A	*0.19* (*0.05; 0.33*)	1.00	*0.39* (*0.28; 0.49*)	1.00	*0.20* (*0.06; 0.34*)	1.00
Parity B	*0.31* (*0.19; 0.43*)	1.00	*0.30* (*0.22; 0.39*)	1.00	− 0.03 (− 0.15; 0.09)	1.00
Parity C	*0.37* (*0.18; 0.55*)	1.00	0.08 (− 0.03; 0.18)	1.00	− *0.20* (− *0.37;* − *0.03*)	1.00

Italic characters indicate estimates, which differ significantly from zero

*z.F*_*L*_ effect of the standardised inbreeding coefficient of the litter, *z.F*_*D*_ effect of the standardised inbreeding coefficient of dams, *z.F*_*A-BL*_ effect of the standardised ancestral inbreeding coefficient of the litter, *Season* effect of the of kindling season compared to summer, *Parity A* effect of the second parity compared to the first parity, *Parity B* effect of parities 3–10 compared to the first parity, *Parity C* effect of parities 11+ compared to the first parity, *RI* relative importance

**Table 4:** Standardised estimates of effects on the survival rate of kits at birth after averaging of models that satisfy the ΔAICc ≤ 2.0 criterion in the in the Kalinowski modelling approach

Variable	Dec 1992 to Aug 1997		Sept 1997 to Oct 2007		Nov 2007 to Nov 2017	
	Estimate (95% CI)	RI	Estimate (95% CI)	RI	Estimate (95% CI)	RI
Intercept	3.20 (2.99; 3.40)	–	2.82 (2.43; 3.20)	–	3.81 (2.75; 4.88)	–
z.F_NEWL_	**− ***0.09* (**− ***0.13;***− ***0.04*)	1.00	0.01 (**− **0.02; 0.02)	0.19	0.03 (**− **0.02; 0.08)	0.26
z.F_NEWD_	0.00 (**− **0.08; 0.07)	0.27	**− **0.04 (**− **0.08; 0.04)	0.46	**− **0.01 (**− **0.03; 0.03)	0.12
z.F_A-KL_	*0.08* (*0.01; 0.17*)	1.00	0.03 (**− **0.02; 0.09)	0.39	0.06 (**− **0.06; 0.09)	0.30
Season	**− ***0.26* (**− ***0.36;***− ***0.16*)	1.00	**− ***0.12* (**− ***0.19;***− ***0.05*)	1.00	**− ***0.62* (**− ***0.71;***− ***0.53*)	1.00
Parity A	*0.20* (*0.06; 0.33*)	1.00	*0.39* (*0.28; 0.49*)	1.00	*0.20* (*0.06; 0.34*)	1.00
Parity B	*0.31* (*0.19; 0.43*)	1.00	*0.30* (*0.22; 0.39*)	1.00	**− **0.03 (**− **0.15; 0.08)	1.00
Parity C	*0.37* (*0.19; 0.56*)	1.00	0.07 (**− **0.03; 0.18)	1.00	**− ***0.20* (**− ***0.37;***− ***0.03*)	1.00

Italic characters indicate estimate that differ significantly from zero

*z.F*_*NEWL*_ effect of the standardised new inbreeding coefficient of the litter, *z.F*_*NEWD*_ effect of the standardised new inbreeding coefficient of the dams, *z.F*_*A-KL*_ standardised Kalinowski (ancestral) inbreeding coefficient of the litter, *Season* effect of the of kindling season compared to summer, *Parity A* effect of the second parity compared to the first parity, *Parity B* effect of parities 3–10 compared to the first parity, *Parity C* effect of parities 11+ compared to the first parity, *RI* relative importance

### Inbreeding depression and purging

In the first period, the model that included only parity, season and animal effects did not meet the 2AIC_C_ criterion. In contrast, the effects of inbreeding were always included in the top models, which confirmed the importance of ID in modelling birth survival (Table 2). In the second and third periods, the model that included parity, season and animal effects was always selected as the best model, although models that included various inbreeding coefficients also met the 2AIC_C_ criterion (Table 2). The effects of dam inbreeding (F_D_ and F_NEWD_) were included in the top models for all periods and for both modelling approaches, although the effects were not significant; their relative importance was 0.19 in the Ballou-Boakes approach (Table 3) and 0.28 in the Kalinowski approach (Table 4).

For all three periods, the top models included at least one ancestral inbreeding coefficient, based on both the Ballou and the Kalinowski approaches, which supports the inclusion of such effects in the model (Table 2). The relative importance of the effect of ancestral inbreeding was greater in the first period than in the subsequent two periods, particularly when using Kalinowski’s ancestral inbreeding coefficients (Tables 3 and 4).

Litter inbreeding (F_L_ and F_NEWL_) affected birth survival significantly in the first period, during which its effects were negative, but it did not significantly affect survival in the second and third periods (Tables 3 and 4). The best model in the first period resulted in significant negative estimates for new inbreeding (z.F_NEWL_ = − 0.09) and positive Kalinowski ancestral inbreeding coefficients effects for litter (z.F_A-KL_ = 0.08), providing additional evidence of ID and its purging (Tables 2, 3 and 4).

## Discussion

### Inbreeding depression and birth survival

#### Effects of litter inbreeding

In the first period, litter inbreeding affected birth survival significantly, with F_L_ and F_NEWL_ showing similar negative standardised effects (Tables 3 and 4). Estimates of z.F_NEWL_ represent ID caused by autozygosity, which occurs in an individual for the first time. These results provide additional evidence of ID in mammalian populations.

Comparing estimates of ID from our study with those reported in other mammalian populations is difficult, since few studies have reported new inbreeding ID. Significant negative impacts of F_NEWL_ were reported for milk production, fertility, health and stillbirth in dairy cattle populations [35, 48, 49]. Our results are consistent, in principle, with a study of Spanish rabbit lines that reported significant ID on the total number of kits born and the number of kits born alive [50]. In that study, ID resulted from new (recent) inbreeding, but not from old (remote) inbreeding.

#### Effects of dam inbreeding

The effects of various dam inbreeding coefficients (F_D_ and F_NEWD_) were always included in the top models (Table 2), which justifies their inclusion in the model. Nevertheless, the effects of dam inbreeding were never significant, suggesting that these effects were too small to be detected with the classical methods applied here. Our results contrast with those of previous studies on other mammalian populations, which documented negative effects on reproduction traits such as litter size or number of stillborn animals in polytocous species such as mice [32], pigs [33], and rabbits [30]. In our previous work on the same population of Pannon White rabbits from 1992 to 2009 as the current study, dam inbreeding increased the number of stillborn kits significantly, whereas litter inbreeding reduced the number of kits born alive [30]. Although that analysis led to an ID estimate that was in the same direction than that in our study, the significant effect of dam inbreeding on the number of stillborn kits in [30] may have been diluted here because we examined a slightly different variable, i.e. survival of kits at birth.

Our previous study on Pannon White rabbits [30] and previous work on other mammalian populations [30, 32, 33] highlight the need to take litter and dam inbreeding into account simultaneously and reflect that the effects of these two types of inbreeding operate via different mechanisms. Falconer [51] noted that litter inbreeding may reduce the viability of embryos and foetuses, whereas dam inbreeding may reduce female fertility. Dam inbreeding may affect the maximal number of foetuses that the uterus can support, independently of the ovulation rate [52]. The contribution of dam inbreeding to ID can be difficult to quantify because of the interplay between uterine capacity and ovulation rate or genetics of the litter (e.g. that increase the rate of early embryonic death).

### Purging of detrimental load

We identified evidence of significant detrimental load purging during the first period in the form of a significantly negative effect of F_NEWL_ and a significantly positive effect of F_A-KL_ (Table 4). ID was not detectable in the two subsequent periods. However, during the first period, Ballou’s ancestral inbreeding coefficients did not indicate purging. In the first period, ID may have occurred through two mechanisms, one captured by the effects of F_NEWL_ and involving several genes with large harmful effects, and another captured by the effects of F_L_ and involving mildly deleterious genes that contribute to polygenic dominance and overdominance. In the second and third periods, the first mechanism may have already been purged, while the second mechanism disappeared or was present but undetectable. This would explain why the effects of F_L_ and F_NEWL_ were not significant in the second and third periods, and why the effects of inbreeding coefficients were considerably less important in those periods than in the first period.

Purging is thought to occur more in populations in which ID is caused by deleterious recessive genes with large effects, when inbreeding occurs gradually over several generations, and when the population is under strong selection [3, 53]. The population of Pannon White rabbits that we studied here satisfies the criterion of gradual inbreeding over a long period: by the end of the study period, more than 60% of the genomes in the population had experienced inbreeding at least once (Fig. 2). This level of ancestral inbreeding is substantially higher than the 6.5 to 10.0% reported in dairy cattle populations [35, 48, 49]. Such high ancestral inbreeding likely helped purge ID because of large harmful mutations. At the same time, the level of new inbreeding [14] remained relatively low and constant throughout the study period (Fig. 2).

Consistent with ID purging in our Pannon White population during the first period, 59% of the founders that contributed to the population disappeared, while the remaining 41% of founders persisted through the rest of the study period (Fig. 1). The strong decrease in the number of founders at the beginning of the study period may reflect natural selection or artificial selection, since artificial selection was never explicitly performed based on survival of kits at birth. Such involuntary selection pressure may have eliminated founders with a higher detrimental load, thereby enhancing ID purging. A study of the Habsburg dynasty in humans showed strong ID purging with respect to child survival within only 10 generations [54]. A similar purging tendency, in which the ID observed in the first period decreased in subsequent periods, was reported in a captive gazelle population [55].

Evidence of ID purging has also been detected in several cattle populations [35, 48, 49] for birth weight, stillbirth rate, milk yield, and milk protein level. Similarly, purging has been described in Sumatran tigers for neonatal survival rate [3], and in a laboratory mouse population for litter size and litter weight [32]. A meta-analysis found that ID declined progressively with time in several populations, which suggests that purging may have had a slightly positive impact [15]. However, whether those studies truly detected purging is called into question by the fact that they generally did not observe significant positive effects of ancestral inbreeding [3, 48, 56]. We agree with previous assessments that ID purging cannot be practically implemented, thus intentional inbreeding of animals is not recommended for purging inbreeding load [8, 57].

### Assumptions, limitations, and perspectives

#### Confounding effects of environment and other factors on the estimation of inbreeding depression

ID is more severe in harsh environments [28, 29, 58], thus environmental improvement in long-term experiments can reduce ID, which may be falsely interpreted as purging. To our knowledge, climate conditions and husbandry practices for the population used in our study remained fairly constant. While artificial selection directed at improving survival at birth has never been applied to Pannon White rabbits, it is possible that indirect selection for growth or body composition traits could interact to alter reproductive traits. For example, Szendrő et al. [59] reported that selection for thigh muscle volume using in vivo computer tomography led to smaller body fat depots, leading in turn to lower reproductive performance. Unfavourable genetic correlations between litter weight on day 21 and thigh muscle volume have been observed in Pannon White rabbits [60], with moderate correlation estimates for parities one (− 0.37) and two (− 0.37), but high estimates for parities three (− 0.53) and four (− 0.70). Detrimental alleles that might interact with the environment and cause ID may have been purged in the first breeding period, which is a known difficulty in obtaining clear evidence of purging [28, 29, 58].

Nutrition is another potential confounder in our analysis of ID and purging. Our animals were given feed from an international supplier from 1992 to 2010, feed from a Hungarian supplier from 2010 to 2013 and from 2016 until the end of the experiment, and feed from a second Hungarian supplier from 2013 to 2015. Although all feeds were labelled with similar nutrient compositions, we cannot exclude that the changes in diet may have influenced ID. Another possibility is that environmental factors that we did not assess or control may have contributed to ID in our rabbit population.

Nevertheless, we believe that confounding by any of these factors is likely to be minimal because ID purging only occurred within the first period that we examined, which seems too short for considerable environmental changes to occur.

#### Need for genomic estimation of inbreeding depression

While significant ID was observed in the first period, we were unable to make any confident conclusions about the genetic processes that underlie the observed ID, although the results from different models suggest a combined impact of large harmful effects and mildly deleterious polygenic effects. To analyse ID and its purging in more detail, it may be necessary to move beyond pedigree data to genomic data. High-throughput sequencing data can provide more accurate estimates of individual inbreeding than pedigree-based analyses for both humans [61] and animals [62, 63]. Indeed, replacing pedigree-based inbreeding coefficients with genomic estimates can estimate ID more accurately [64–66] and identify specific loci that contribute considerably to ID [67, 68]. Unfortunately, currently we lack the necessary genomic information to perform such analyses in this population, but these should be considered in future work.

## Conclusions

Inbreeding depression for survival of kits at birth was observed in the first part (1992–1997) of a 25-year study period (1992–2017) in a closed Pannon White rabbit population. Litter inbreeding contributed significantly to models with litter inbreeding (F_L_) and models with new inbreeding coefficient of the litter (F_NEWL_), indicating a complex genetic architecture for inbreeding depression. Evidence of inbreeding depression purging was observed in the form of negative effects of litter inbreeding coefficients and positive effects of the Kalinowski ancestral litter inbreeding coefficients for the period 1992–1997. The speed of purging suggests that confounding due to environmental changes is not likely, although we cannot exclude it entirely. Our approach was based on information theory and multi-model inference, and it was implemented using generalized linear mixed models that accounted for polygenic random effects and that relied on the logit link function. This approach may be useful for further studies to clarify under what conditions and via what genetic mechanisms purging of detrimental load can occur in domesticated mammalian populations.

## Data Availability

After acceptance of the manuscript, all related data (phenotypic and inbreeding records, together with R-scripts used in analyses will be archived in the institutional repository of the Kaposvár University.
